# Interpreters as Vital (Re)Tellers of China’s Reform and Opening-Up Meta-Narrative: A Digital Humanities (DH) Approach to Institutional Interpreters’ Mediation

**DOI:** 10.3389/fpsyg.2022.892791

**Published:** 2022-07-11

**Authors:** Chonglong Gu

**Affiliations:** Department of Chinese and Bilingual Studies, The Hong Kong Polytechnic University, Kowloon, Hong Kong SAR, China

**Keywords:** political press conferences, interpreter mediation, knowledge (re)construction, political knowledge, corpus-based CDA, East-West power differentials, Reform and Opening-up meta-narrative, digital humanities

## Abstract

If the important role of written translation in the construction and contestation of knowledge and narratives remains largely under-explored, then the part played by interpreting and interpreters is even less examined in knowledge construction and story-telling. At a time when Beijing increasingly seeks to bolster its discursive power and have the Chinese story properly told, the interpreter-mediated and televised Premier-Meets-the-Press conferences constitute a typical discursive event and *regime of truth* in articulating China’s officially sanctioned “voice.” Discursive in nature, the institutionalised event permits Beijing to construct a desired version of truth, fact and narrative in front of the more vociferous and dominant West. As an attempt to employ digital humanities (DH) methods to real-world language use, this corpus-based CDA study explores the press conference interpreters’ agency and mediation in rendering key concepts and discourses (e.g., ECONOMY and REFORM) constitutive of the broader Reform and Opening-up meta-narrative, which legitimises China’s political and economic systems, developments, policies and positions in the post-1978 era. The interpreters are found to maintain and often further reinforce Beijing’s discourse in English at various levels. Apart from indicating issues of institutional alignment, this study points to interpreters’ agency in communicating beyond national borders, (re)constructing political and institutional knowledge, counterbalancing the naturalised and taken-for-granted Western narratives, and contributing to the shifting East-West power differentials discursively. This study highlights the significance of interpreters as indispensable (re)tellers of the Chinese story in an increasingly mediat(is)ed world, where the interpreted discourse is routinely quoted *verbatim* on various platforms and taken for granted as the correct version of Beijing’s official “voice.”

## Introduction

The various encounters with the Western imperial powers (signalled by the Sino-British Opium War in 1840) led to China’s gradual degeneration into a “semi-colonial and semi-feudal” society. After the “century of humiliation,” “new China” was born in 1949 under the Communist Party of China, pursuing a path of socialism. After a few eventful decades of exploration, Deng Xiaoping initiated the reform and opening-up programme in 1978, where China has embarked upon a road of “socialism with Chinese characteristics.” After decades of rapid development, China has now become the 2nd largest economy and the biggest developing country in the world. In addition to the concrete developments on various fronts, Reform and Opening-up forms an overarching (meta)discourse, justifying and legitimating China’s sociopolitical systems and miscellaneous policies since 1978, both domestically and in front of the international community (Ploberger, 2016; Shen, 2018; Gu, 2022). Against the backdrop of reform and opening-up, the interpreter-mediated premier’s press conferences were established as a televised annual event. In this institutionalised discursive event, the Chinese premiers answer domestic and international journalists’ questions (Gu, 2019a, b) and articulate China’s versions of fact, truth and reality on various topics (China’s economic development, the government’s financial policies, domestic reforms, trade frictions, people’s livelihoods, agriculture, China-Japan ties, Beijing’s positions on Taiwan and Tibet, etc.). However, despite the wide range of domestic and international topics covered, the recurring theme of reform and opening-up constitutes a mainstay of the conferences, cutting across different facets of China’s everyday existence and management.

There are studies galore in the political sciences and economics about China’s market-oriented reform (Coase and Wang, 2012; Ploberger, 2016; Shen, 2018). However, there is a lack of analysis in academia from a discursive perspective relating to how Beijing’s guiding reform and opening-up (ROU) discourse is articulated. Particularly, there is a gap in how the ROU discourse is articulated and mediated in China’s international language, that is, English by government-affiliated interpreters (who are usually communist party members and civil servants recruited into China’s Ministry of Foreign Affairs). Similarly, within critical discourse studies, while there have been numerous studies on monolingual discursive communication, relatively few studies have explored bilingual discursive communication, that is, translation and interpreting. More recently, there have been a limited but growing number of studies that focus on translation and ideology on various platforms and across different languages and genres (Hatim and Mason, 1990; Kang, 2007; Munday, 2007; Valdeón, 2007; Zhang, 2013). However, in contrast, there are very few CDA-informed studies investigating interpreters’ discursive and ideological mediation. Amongst these, most empirical studies so far have focussed on only a few individual linguistic features and discursive categories such as modality, personal pronouns, ideologically salient lexical items, and metadiscursive markers (cf. Beaton-Thome, 2013; Gu, 2018; Li, 2018; Gu and Tipton, 2020) without due attention to the propositional content and without investigating a sustained narrative or discourse.

A socially shaping discourse, the interpreted version of China’s ROU discourse has an international dimension, serving to shape opinions and facilitate the proper telling of China’s story (*jianghao zhongguo gushi*) in front of a global audience. This highlights the potential importance of interpreting in getting China’s discursive and ideological message across beyond China’s national borders and contributing to the shifting power differentials between the global North and South and the East and West. Framed within the broader backdrop of *jianghao zhongguo gushi* and perceiving the ROU as a fundamental overarching meta-narrative justifying and pervading China’s development over the past few decades, this article, as part of a series of empirical studies, explores the interpreters’ mediation of China’s ROU meta-narrative in English. That is, in terms of research questions, the study aims to establish how key concepts and discourses central to China’s ROU are rendered cross-linguistically at a lexical level overall and more specifically how the constitutive ECONOMY and REFORM discourses are (re)presented and mediated by the government-affiliated interpreters. If interpreter agency is found, what might be the discursive effect or impact of this globally. Benefitting from the recent technological affordances and harnessing a digital humanities methodology, this corpus-based CDA study promises to shed light on the interpreters’ agency in (re)articulating the voice of the world’s largest developing country in our increasingly mediat(ise)d world.

## Reform and Opening-Up as an Overarching Meta-Narrative in Post-1978 China

Metadiscourses or meta-narratives (or French *grands récits)* are grand and dominant “big stories,” which play an overarching role in structuring and legitimising other smaller constitutive stories and related events. Throughout human history, there have been various such metadiscourses such as Enlightenment, democracy, capitalism, and, more recently, “war on terror” and “the clash of civilisations.” In contemporary China, there are two major overarching discourse modes, that is, revolutionary discourse (1949–1977) and reform discourse since 1978 (Gu, 1996). After a few eventful decades of close-door socialist experimentation since the founding of the People’s Republic of China in 1949, Beijing finally decided upon a road of “Socialism with Chinese Characteristics.” This change in discourse mode reflects a major ideological shift toward a pragmatist approach, focussing on reform and economic development. A centrepiece of this is the reform and opening-up programme initiated by Deng Xiaoping in 1978 - a pragmatic *modus vivendi* which involves incorporating elements of capitalism to China’s socialist system. Now, around 45 years on, despite the setbacks and challenges (e.g., recent trade war), China has registered years of world-beating economic growth and is poised to overtake the United States as the largest economy in the world, under the formula of economic reform. A superpower-in-waiting and the largest and arguably most successful developing country in the globe, China and its rise are bound to change the dynamics between the global north and south.

Like Marxism, “the clash of civilisations,” and multiculturalism, “Reform and Opening-up” constitutes one of the major macro-level meta-narratives (“grands récits”) in post-1978 China, which consists of such constitutive discourses and narratives as economy, development, market, reform, stability and openness (Ploberger, 2016; Shen, 2018; Gu, 2022). Under each constitutive discourse or theme, there are also numerous instances and interplay of micro-level policies, decision-making and individual events (e.g., China’s entry into WTO, the management of exchange rates, market entry for foreign companies, and more recently the US-China trade war). Such an overarching formula and its associated discursive articulations justify Beijing’s various policies and strategies on the economic and sociopolitical fronts, witnessing China’s rapid development over the past few decades. The ROU therefore constitutes a major meta-narrative, permitting China to have its voice heard in the international community as the world’s largest developing country.

## Theoretical Framework, Data and Methodology

Rather than looking at narrative and discourse as completely unrelated concepts, this study views China’s broader ROU meta-narrative as being realised via various constitutive elements or discourses. Given the political and discursive nature of this event and the aims of this study, Critical Discourse Analysis (CDA) would provide a suitable theoretical framework. Having emerged from “critical linguistics” developed at the University of East Anglia, Critical Discourse Analysis (CDA) is an interdisciplinary and socially oriented approach to the study of discourse that views language as a form of social practice. It examines how discursive sources are maintained and reproduced within specific social, political, institutional, and historical contexts. As a socially engaged and problem-oriented approach, CDA is concerned with exposing and making more explicit the otherwise opaque or hidden ideologies and/or latent power asymmetries enacted, legitimised and reproduced in discourse (Fairclough, 1989). For CDA scholars, discourse is both socially shaped and socially constitutive, thereby possessing the ability to shape reality and effect change. Notably, far from a monolithic and homogeneous approach, there are various schools in CDA (e.g., Fairclough, 1989; Wodak, 2001; van Dijk, 2008). However, despite its multifarious and eclectic nature, all CDA approaches arguably are united by the shared take-nothing-at-face-value critical attitude and the ultimate agenda to reveal power and ideology embedded in discourse at various levels.

So far, CDA is mostly applied on monolingual texts (e.g., newspapers, political manifestos, and public speeches). Given the bilingual and mediated nature of the premier’s press conferences, interpreting is conceptualised as a (re)contextualisation process at a macro level, with the interpreter serving as the intertextual and interlingual connecting point between the source text (Chinese) and the target text (English). The fact that information is inevitably rendered into the sociopolitical, cultural and linguistic contexts of the TT highlights the possible micro-level decision-making, stance-taking and possibly ideological mediation that occur in the interpreting process. This macro-level conceptualisation permits a critical comparative analysis between the Chinese ST and English TT, focussing on ideologically salient shifts.

Traditionally, CDA is qualitative in nature, featuring close manual analysis of a small sample of text. However, despite its apparent advantages of being in-depth, the traditional qualitative CDA is subject to criticism (cf. Widdowson, 1995; Orpin, 2005) that the qualitative analysis is often not systematic, objective and representative enough and practitioners might sometimes cherry-pick information to suit their own worldviews and ideological agendas. Therefore, corpus linguistics (CL) is increasingly incorporated (Partington, 2004; Mautner, 2009; Baker, 2012) to reduce researcher bias.

For more systematic and objective analysis, a mixed-methods digital humanities approach of corpus-based CDA is employed in this empirical study, thus permitting a dynamic triangulation between the typically qualitative (CDA) and the typically quantitative (CL). Given the overriding need to conduct more **data-driven** study to guide the analysis using the corpus data, CDA here, as with most similar studies, is used as a general source of theoretical insight, without limiting itself to or rigidly following any particular school. Having said that, once ideologically salient patterns or interesting phenomena are found, relevant concepts, notions and discursive devices mentioned by key CDA scholars (e.g., van Dijk and Fairclough) as well as relevant CDA studies will be invoked to help interpret the data, explain the phenomena and enrich the otherwise quantitative analysis. The corpus-based CDA analysis draws on the CE-PolitDisCorp (Chinese-English Political Discourses Corpus) established by the author for investigating the various aspects of China’s political interpreting and discourses in Chinese and English. The CE-PolitDisCorp consists of 20 years of China’s Premier-Meets-the-Press conference data (1998–2017). The bilingual corpus contains 310,924 tokens in total (170,260 tokens in Chinese and 140,664 tokens in English). A more detailed breakdown of the corpus data is presented in Table 1. On average, one press conference lasts for approximately 2 hours. Since there is one press conference each year, there are, in total, 20 press conferences in the CE-PolitDisCorp, spanning over three latest administrations so far: Zhu (1998–2002), Wen (2003–2012), and Li (2013–2017). The essentially diachronic nature of the corpus data makes it possible to identify consistent and relatively stable patternings over time. Since the main body of the data involves (1) the Chinese premiers’ utterances in Chinese (subcorpus A) and (2) their corresponding interpretations into English (subcorpus B), these two components form the main focus of this current digital humanities (DH) study, drawing on corpus-based critical discourse analysis.

**TABLE 1:** A detailed breakdown of the various subcorpora of the CE-PolitDisCorp.

CE-PolitDisCorp (1998–2017)			
Subcorpus A	Subcorpus B	Subcorpus C	Sub-corpus D
China’s official discourse in Chinese	China’s interpreted discourse in English	China’s official discourses in Chinese and English (sum of sub-corpus A and B)	Journalists’ questions and their respective interpretations
127,696 tokens	105,495 tokens	233,191 tokens	77,733 tokens

It is worth noting that transcripts of the premier’s press conferences are available on China’s government websites. However, the official transcripts have been extensively edited and, therefore, often do not accurately reflect the Chinese premiers’ and the government-affiliated interpreters’ precise utterances. The corpus data, as such, was transcribed *verbatim* from videos available on China’s official websites as well as on video-sharing sites such as YouTube and Youku meticulously. Understandably, given the intrinsically evanescent nature of spoken utterances (Shlesinger, 1998, 4), the data collection and preparation processes are highly labour-intensive and time-consuming, involving considerable efforts. The fully prepared data (e.g., after segmentation for Chinese) is analysed using the AntConc software (3.4.4 windows), a freeware developed by Laurence Anthony at the Waseda University. This corpus linguistics software contains a range of functions including concordancing, wordlist generation (lexical frequency), keyword generation, Kwic sorting, and other tools for studying clusters/N-grams, regular expressions (REGEX) and collocations. Also, the Antconc software is Unicode compliant, making it possible to work on most languages (including Chinese and Korean). More details on the corpus data and software used can be found in Gu (2018, 2019c).

## Data Analysis

Attention is first focussed on the interpreters’ rendering of China’s broader ROU meta-narrative at an overall lexical level, before exploring the constitutive economy and reform discourses in more detail drawing on corpus-based CDA.

### Interpreters’ General Level of Mediation on Constitutive Concepts and Themes

As discussed previously, the overarching reform and opening-up (ROU) meta-narrative is inevitably realised in the form of various major concepts and broader themes. This section focuses on the interpreters’ general mediation on various concepts and themes. After applying AntConc’s frequency list function, various top lexical items can be established (given the limited space the full list will not be provided here). These individual lexical items can be subsumed under the following major concepts and themes that are *directly* related to ROU: DEVELOPMENT, ECONOMY, REFORM, INTERNATIONAL ENGAGEMENT AND GLOBAL INVOLVEMENT, MARKET, STABILITY, OPENNESS, MODERNISATION, SOCIALISM, and HARMONY. Please see Table 2 for a more detailed breakdown. Other frequent items such as “we” (2,103 instances), “China” (1,076 instances) and “people” (699 instances) may not be directly related to Reform and Opening-up and, therefore, will not form the main focus here. These concepts identified above can be seen as more concrete thematic realisations and different constitutive facets of the ROU meta-narrative. The instances of the various broader concepts in both Chinese and English are provided in Table 2. In establishing the statistics, the wildcard function (*) was used to cover the various lexical realisations of a particular broader concept/discourse in the subcorpora. In addition, in counting the instances, different scenarios were also taken into consideration. For example, in Chinese, words are sometimes formed by compounding and clipping. “经贸” (jing mao), for instance, is a shortened form of “经济贸易” (jingji maoyi or “economy and trade”). Such possibilities were taken into account in the calculation to ensure more accurate results.

**TABLE 2:** Instances of lexical items relating to ROU in Chinese and English subcorpora.

Broader concept/discourse	Chinese subcorpus (freq)	English subcorpus (freq)	Change (%)
DEVELOPMENT	发展* (451)	develop* (539)	+19.5
ECONOMY	经济* (450)	econom* (526)	+16.9
REFORM	改革* (323)	reform*/restructur* (392)	+21.4
INTERNATIONAL ENGAGEMENT/ INVOLVEMENT	全球*/世界*/国际* (302)	glob* (including global, globalise, globalised, globalisation) (362)	+19.87
MARKET	市场* (172)	market (227)	+32
STABILITY	稳* (稳定, 稳定性, 稳健, 稳固, 平稳 *etc.*) (157)	Steady, stable and stabili* (including stability, stabilise, stabilised, stabilising, stabilisation) (156)	−0.64
OPENNESS	开放/放开/敞开/公开 (121)	open* (136)	+12.4
MODERNISATION	现代* (44)	modern* (45)	+2.3
SOCIALISM	社会主义* (42)	socialis* (43)	+2.3
HARMONY	和谐* (11)	harmon* (including harmony and harmonious) (15)	+36.3
**Overall (freq)**	**2,073**	**2,441**	**+17.75**

Such a focus on lexical items is important. That is, the premier’s frequent (or infrequent) articulation of certain important items (*reform*, *economy, Taiwan*, etc.) is believed to reflect the government’s level of attention, which is often widely used by the media and China observers within China and without as a major barometer to decipher Beijing’s changes in policy and shifts in its priorities. This crude overall analysis shows that important concepts central to the ROU meta-narrative are made more prominent in English *vis-a-vis* the Chinese original at a lexical level due to interpreting (a 17.75% increase overall and noticeable increases in most categories). This is salient ideologically and rhetorically and constitutes a case of repetition (Fairclough, 2000; Beaton, 2007; Lahlali, 2012; Gu, 2019c,^2022^). If the (under)production of important lexical items pertaining to China’s ROU metadiscourse would lead to the metadiscourse becoming diluted and weakened, the interpreters’ statistical (over)production of those lexical items points to increased interpreter alignment *vis-a-vis* the government and a further strengthening of Beijing’s ROU discourse and *regime of truth* in a Foucauldian sense overall (Foucault, 1984). From the perspective of discursive effect, the interpreters have facilitated and reinforced the conveyance of the government’s ROU metadiscourse and, by extension, the Chinese story (*zhongguo gushi*) before the global audiences worldwide (see Gu, 2022 for more detailed and focussed discussion).

Considering the importance of the ROU as an overarching meta-narrative permeating different aspects of China’s developments, policies and positions since 1978, the interpreter-mediated discourse works to counterbalance the often naturalised and seemingly commonsensical narratives of the West (Said, 1978; Shi-xu, 2014), thus contributing to the constantly shifting power differentials between geopolitical actors in the world at least discursively. From the perspective of image (re)construction, the interpreters have also (re)constructed a more favourable image of Beijing being more market, economy, and development-focussed, keen on reform and modernisation, increasingly open internationally, and more globally-oriented in its reform and opening-up endeavour. Interestingly, the slightly increased production of items relating to socialism also seems to (re)affirm the fact that such reform and opening-up efforts are essentially socialist in nature (cf. later sections for more details).

Given the limited space, the extensive nature of corpus-based analysis and other practical considerations, it would not be practicable to present robust and detailed discussions on all of those broader concepts in this paper-length article. With the aforementioned statistical information being a useful point of departure, more in-depth, robust and fine-tuned analysis is provided below on two of the top concepts **ECONOMY** (526 instances) and **REFORM** (392 instances) as entry points or “way-ins” into the interpreters’ rendering of the ROU meta-narrative. These two frequently articulated concepts have permeated through the premier’s press conferences both in Chinese and the interpreted discourse in English. This therefore makes these interesting to explore in a more systematic manner.

Discourse is inevitably constructed on many different levels. This corpus-based analysis approaches the various constitutive discourses both at a lexical level overall and in the form of collocations and patterned constructions. On a lexical level, the various forms of certain lexical items are explored and compared in both subcorpora from an overall perspective and diachronically. This constitutes the most straightforward and targeted way of investigating interpreter mediation. Statistically, the more times certain item(s) are (re)articulated in interpreting, the more prominence they are given in the TT (hence a higher level of interpreter alignment *vis-à-vis* the government’s policies and a strengthening of its ideological discourse).

Additionally, discourse is (re)constructed through collocations or the “company” (Firth, 1957, 179) certain items keep. A word’s collocations are “statements of the habitual and customary places of that word” (*ibid.*, 181). Collocations can be of significance either because they are frequently repeated or they are unexpected (Sinclair, 1991). The collocate lists of certain items are generated for both subcorpora and placed into different semantic groups, thus making it possible to shed light on the actual propositional content and identify what items/concepts are made more closely related to the search items and what are given more prominence in the interpreted discourse. To do this, T-score is selected as the collocate measure and the default window span of five words to the left and right of the node word is employed for both subcorpora. Although the optimal window span for investigating collocates remains a matter of dispute, a span of −5 and +5 is commonly adopted and is considered sufficient to retrieve “more than 95% of all relevant information” (Thomas, 1993, 47). As such, an initial window span of five words to the left and right is set as a starting point. If necessary, the span is widened. Throughout the process, attention is placed on interesting patterns discerned in the concordance lines. Where necessary, attention is also paid to the interpreters’ rendition of fixed slogan-like formulations or *tifa* (提法). The pithy and definitive-sounding formulations are condensed distillations of the government’s ideological discourses. Studying the important slogans and fixed constructions can indicate major sociopolitical and ideological changes in the Chinese context and pinpoint the interpreters’ level of mediation. Examples are also provided to illustrate the micro workings of interpreter agency.

### Interpreters’ Mediation of Beijing’s Economy and Reform Discourses

The interpreters’ discursive mediation of the two themes, that is, economy and reform is discussed at various levels in sections “Interpreters’ Mediation of Beijing’s Discourse on the Economy” and “Interpreters’ Mediation of Beijing’s Discourse on Reform.”

#### Interpreters’ Mediation of Beijing’s Discourse on the Economy

A central aspect of China’s pragmatist reform and opening-up is “economy.” This explains, in part, why economic issues often take centre stage in each year’s press conference. It is therefore interesting to examine the interpreters’ mediation of China’s discourse on the economy at the following levels.

##### Overall Level of Mediation of the Economy

To establish the overall level of interpreter mediation on economy-related items, 经济* and econom* were searched in both subcorpora. As Table 3 suggests, the various forms of the word “economy” are mentioned more in the TT both in aggregate terms (a 16.9% increase) and diachronically in each period. The proliferated mentions of econom* constitute a case of lexical repetition (Fairclough, 1989; Beaton, 2007), pointing to an increased level of interpreter alignment with and a further strengthening of the government’s discourse on “economy” overall. This is of particular relevance, given that the Chinese government’s economic performance and the concrete benefits it delivers economically constitute an important source of its achievements and legitimacy (Zhao, 2009, 426).

**TABLE 3:** Econom* in both subcorpora and across the administrations.

	Chinese subcorpus (freq/freq per year)	English subcorpus (freq/freq per year)	Increase (%)
Zhu (1998–2002)	43/8.6	55/11	27.90
	(7082.6 tokens/year)	(6101.6 tokens/year)	
Wen (2003–2012)	275/27.5	319/31.9	16
	(8614.5 tokens/year)	(7166.3 tokens/year)	
Li (2013–2017)	132/26.4	152/30.4	15.10
	(9740.4 tokens/year)	(7698.6 tokens/year)	
Overall	450	526	16.90

Interestingly, the presence of the various forms of the word “economy” has increased over time in both subcorpora. On an annual basis, the numbers rose dramatically in Wen’s administration from relatively low levels in Zhu’s administration, before becoming relatively stabilised in Li’s period. This trend is not surprising, given China’s entry into the WTO in 2001 and the fact that China is now a major driver of global economy after decades of economic reform. In other words, with the deepening of China’s reform and opening-up, “economy” is featured increasingly prominently at these press conferences. Without doubt, a more favourable image of Beijing being (increasingly) focussed on economic development and reform is (re)constructed by the interpreters in front of the global audience.

##### Collocational Patterns Relating to the Economy

For more contextualised analysis, the collocates of 经济* and econom* were generated in both subcorpora and presented based on the following semantic categories (Table 4). A comparison between the collocates for 经济* and econom* shows interpreters’ mediation in English. For example, the social actors that is the Chinese government and the broader WE are made more visible in the interpreted discourse through the extensive additions of self-referential terms (69.8% increase), thus making it even more emphatic that the Chinese government is the vital social actor responsible for China’s economic activities in English.

**TABLE 4:** Collocates of econom* in both subcorpora.

	Chinese subcorpus (freq)	English subcorpus (freq)
Social actors	中国109; 我们66; 政府 7	China 99/Chinese 42;
		we 84/our 75/us 5;
		government 4
Action and change	发展116;保持30;增长29;复苏17; 解决14;推进14;促进14;调整12;稳定12;平稳11;下行11;影响10;支持9;建设9;运行8; 继续8; 开放8;转型7;改善7;推动7;带来7;实现7; 提高6; 增加6;协调6; 升级6;防止5; 转变 5	Development 93/develop 13; growth 81; restructuring 22; steady 13; recovery 13; ensure 10; downward 9; continue 9; transformation 8; achieve 8; stable 6; promote 6; maintaining 6/maintain 5; grow 6; transforming 5/transform 2, managing 5; upgrade 4
Modality	要38;会22;可以12;必须11; 应该 6	will 47; need 11; must 11; can 10; should 8
Other related important concepts	世界39; 改革34; 社会29; 问题 25; 体制25; 香港24; 市场24; 结构 17; 政治 17; 贸易14; 国际14; 压力14; 合作13; 金融11; 速度 11; 目标 11; 困难11; 国家10; 国民; 方式9; 就业9; 实体9; 两岸9; 自由7; 民生7; 文化7; 政策7; 形势 7; 企业7; 财政6; 社会主义 6; 矛盾6; 法治 6	trade 30; social 27; global 27; market 26; world 19; cooperation 19; political 17; Hong Kong 16; structure 15; problem 13; reform 12; country 11; structural 10; socialist 9; regulation 9; real 9; pressure 9; measures 9; international 9; inflation 9; progress 8; financial 8; target 7; system 7; national 7; quality 6; performance 6; momentum 6; efficiency 6; vitality 5; society 5

Regarding modality, the level of commitment and determination is relatively well maintained in the TT. In terms of the propositional content, 经济* and econom* in both subcorpora are strongly associated with *development* and *growth*. This indicates that “economy” constitutes an important area in China’s overall development and the maintenance of steady and reasonably high economic growth (evidenced in such collocates as *ensure*, *steady*, *stable*, and *maintain*) is a core preoccupation for Beijing, given the increasingly performance-based nature of the government’s legitimacy.

To identify more specific patterned constructions, concordance lines containing “econom*” were sorted on both sides in English. Amongst the most frequent patterns are direct references to China’s domestic economy: *Chinese economy* (37 times), *China’s economy* (24 times), *our economy* (16 times), *national economy* (7 times), *real economy* (6 times) and the nature of China’s economy, that is, *market economy* (14 times). These concordance lines are relatively evenly distributed across the 20 years’ data, suggesting that China’s domestic economy has consistently been an important topic on the government’s agenda.

Notably, of the 14 instances of *market economy* in English, nine instances (64.3%) are pre-modified by “socialist.” After the previous endeavours, China started to pursue a “socialist market economy” in the post-1978 era under the rubric of “socialism with Chinese characteristics.” “Socialist market economy,” thus, constitutes a fundamental formulation at the core of China’s economic reform, rationalising the pragmatist shift from the previous centralised planned economy (1949–1978) to one that harnesses the power of market in resource allocation (traditionally deemed capitalist rather than socialism). This slogan-like formulation “socialist market economy” and its closely associated formulation “socialism with Chinese characteristics” represent the central watchwords of China’s political and economic development, constituting a crystallisation of all the reforms, initiatives and ideological formulations in the post-1978 period. The overarching formulations highlight that the application of socialism in the Chinese context needs to take into account China’s unique national conditions. The underlying rationale is that China is very much in the “primary stage of socialism” and there is a need to incorporate elements of market techniques in order for China to develop. Such economic pragmatism is cited as essentially a form of “state capitalism” (Bremmer, 2010) and there is believed to be structural contradiction between China’s (capitalistic) market-oriented economic reform and its ideological formations as a socialist country. “Socialism with Chinese characteristics,” nevertheless, provides sufficient “discursive space” (Chen, 2005, 46) and justification for China’s pragmatist market-oriented reforms under Communist rule. Such pragmatism is epitomised in Deng Xiaoping’s audacious and explorative learn-as-you-go “crossing the river by feeling the stones” (*mezhe shitou guohe*) approach and his famous cat metaphor that “it does not matter whether it is a black cat or a white cat, as long as it catches the mice it is a good cat” (*buguan heimao baimao zhuadao laoshu jiushi haomao*). Such a pragmatic and flexible approach has facilitated China’s sustained economic growth and unprecedented rise in recent decades. Close critical comparison shows that the central formulation “socialist market^1^ economy” is rendered more visible in English (6 mentions of 社会主义市场经济 in the Chinese ST *versus* 9 in the English TT), hence a 50% increase. Therefore, China’s justificatory discourse relating to its economic system is further legitimised and justified in the interpreted discourse. This is evidenced in Example 1, where the interpreter has added “under the socialist market economy” and made Beijing’s official economic policy more prominent in English. Discursively, this helps further foreground the essentially *socialist* nature of China’s (relatively successful) economic development and works to (re)tell China’s story in an emphatic manner in front of the global audience.

**Example 1 (2001)**

**ST:** 所以在现在我们政府的职能, 我刚才讲了, 就是要转变为代表国家来对市场进 行监督 , 查处, 保护消费者和人民的利益。

**Gloss:** So, now, our government’s function, as I said earlier, is to shift toward representing the country to supervise the market, to investigate and punish and protect the interests of the consumers and people.

**TT:** The function of the government under the socialist market economy is to properly supervise over the market operations, strengthen regulation, fight against those shoddy goods and fake products and to protect the rights and interests of the consumers and of the people.

**FIGURE 1:**
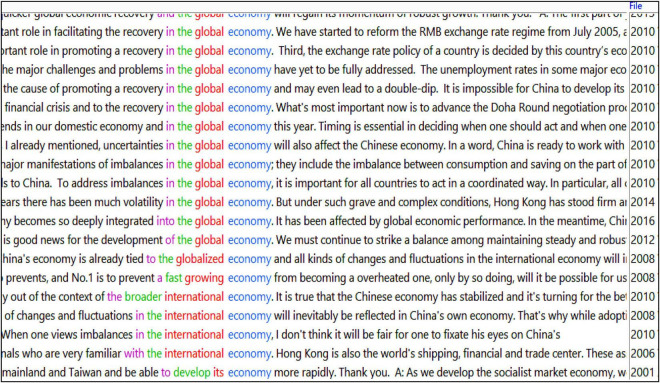
Screenshot of concordance lines featuring global(ised)/international economy (sorted to the left).

Also, Beijing’s discourse on economy goes beyond China’s domestic context and has an international dimension. Interestingly, such collocational patterns as *global economy* (14), *world economy* (9), *international economy* (4) and *globalised economy* (1) began to surface around 2008 in the English subcorpus, roughly coinciding with the global financial crisis started that year (Figure 1).

This sudden uptick in the references to global/world/international economy marks a noticeable shift in China’s official economy discourse from one that was largely inward-looking (focussing on domestic economic policies and initiatives) to one that is increasingly internationally-oriented. This is unsurprising given that China, relatively unscathed by the economic downturn, became a major engine for world economic growth and overtook Japan as the world’s second largest economy in the aftermath of the financial crisis. Notably, China’s economic growth started to slow down gradually around 2015, entering into what is officially called a “new normal.” At a time when the Chinese economy is in need of restructuring, mentions of *new economy* (three instances) and *sharing economy* (one instance) start to appear in the 2016 and 2017 conferences as potential new drivers of China’s economic growth. Close comparisons between the TT and ST show that the patterns and trends (re)presented in English are accurate reflections of the Chinese originals, hence a general level of interpreter alignment and general maintenance of the discursive message in Chinese.

#### Interpreters’ Mediation of Beijing’s Discourse on Reform

Without doubt, reform constitutes a key component of the reform and opening-up programme. To explore the interpreters’ discursive mediation, China’s discourse on reform is approached at the following levels:

##### Overall Level of Mediation on Reform

For a lexical level comparison overall, 改革* and reform*/restructur* (both are used to render 改革 in the Chinese ST) were searched in subcorpus A and B, respectively. Reform*/restructur* are found to be more prominent in the interpreted discourse than their Chinese counterpart both in absolute terms (21.4% increase) and diachronically in each period (Table 5). Such increased (re)production (*cf.* Example 2) again constitutes a case of repetition (Fairclough, 2000; Beaton, 2007; Lahlali, 2012). This signals an increased level of interpreter alignment overall, reinforcing China’s reform discourse and (re)constructing a more positive image of the government being reform-minded in front of the global audience.

**TABLE 5:** Reform*/restructur* in both subcorpora and across the administrations.

	Chinese subcorpus	English subcorpus	Increase (%)
	(freq/freq per year)	(freq/freq per year)	
Zhu (1998–2002)	68/13.6 (7082.6 tokens/year)	76/15.2 (6101.6 tokens/year)	11.8
Wen (2003–2012)	183/18.3 (8614.5 tokens/year)	214/21.4 (7166.3 tokens/year)	16.9
Li (2013–2017)	72/14.4 (9740.4 tokens/year)	102/20.4 (7698.6 tokens/year)	41.7
Overall	323	392	21.4

Similarly, the interpreters’ increased (re)production of reform*/restructur* has become more pronounced progressively over the three administrations (11.8%, 16.9%, and 41.7%, respectively). From a product-oriented perspective, this conveys a heightened sense that, as China’s reforms deepen, the Chinese leadership is increasingly reform-minded and determined to go ahead with various reforms. As such, the interpreter-mediated discourse is conducive to the articulation of the government’s “regime of truth” (Foucault, 1984) in the global language, that is, English.

##### Collocational Patterns Relating to Reform

For a more refined analysis, the collocates of 改革* and reform*/restructur* were established in both subcorpora (excluding such items as *of*, *a*, *in*, *and*, *to*, *is*). The collocates were then categorised into the following semantic groups (Table 6).

**TABLE 6:** Collocates of reform*/restructur* in both subcorpora.

	改革*	reform*/restructur*
	Chinese subcorpus (freq)	English subcorpus (freq)
Social actor (self-referential items)	我们(68); 中国(42); 政府(26);	We (93)/our (48)/us (4); China (51); government (38)
Action and change	推进(53); 进行(23); 推动(10); 继续(8); 深化(8); 实行(8); 建立(7); 完善(7); 解决(6); 坚持(6); 进一步(5); 形成(5); 解放(4); 促进(4); 加快(3); 加强(3); 调整(2); 稳定(2); 确保(2); 激活(2); 前进(2); 着力(1);	pursu* (27); advanc* (13) press (ahead with) (12); deepen* (12); continue (10); conduct* (8); promote (7); further (7); forward (7); introduce* (7); start* (6); endeavour* (6); push* (6); streamlin* (5); establish* (4); launch* (4); achieve* (3) complet* (3); supply (3); stimulate (1); stabilised (1); energise (1); accelerate (1)
Modality	要(46); 必须(8); 会(8); 应该/应当(6); 需要(6); 可以(4)	Will (56); need (19); must (11); can (10); should (8), have to (2)
Other related important concepts and propositional content	体制(86); 制度(44); 农村(35); 经济(34); 政治(33); 开放(28); 金融(22); 建设(18); 汇率(16); 发展(15); 企业(15); 机构(12); 社会(11); 人民币(11); 成功(11); 机制(10); 市场(10); 国有(10); 银行(9); 财税(8); 粮食(8); 人民(8); 结构(7); 管理(7); 税费(7); 司法(7); 医疗(7); 财政(6); 社会主义(6); 目标(6); 国家(6); 领导(5); 过程(5); 行政(5); 简政放权(5); 结构性(5); 商业(5); 税收(4); 现代化(4); 市场化(4); 创新(4); 政策(3); 投资(3); 进程(1); 进展(1);	System(s) (64); political (36); economy/economic (35); opening (up) (28); rural/countryside/agriculture (28); structural/structure (19); development/develop (18); market (14); exchange rate (14); financial (13); state (12); tax (11); enterprise(s) (11); step(s) (10); RMB (9); institutional (9); measure(s) (8); effort(s) (8); grain(s) (8); fee (7); distribution (7); interest(s) (7); regime (7); taxation (6); banking (6); social/society (6); people (6); initiative(s) (6); sector (5); process (5); commercial (5); education(al) (5); task (4); success (4); modernisation (4); innovation (4); governance (4); goal (4); banks (4); socialis* (4); income (3); progress (3); management (3); stability (2); adjustment (2); invest/investment (2)

**FIGURE 2:**
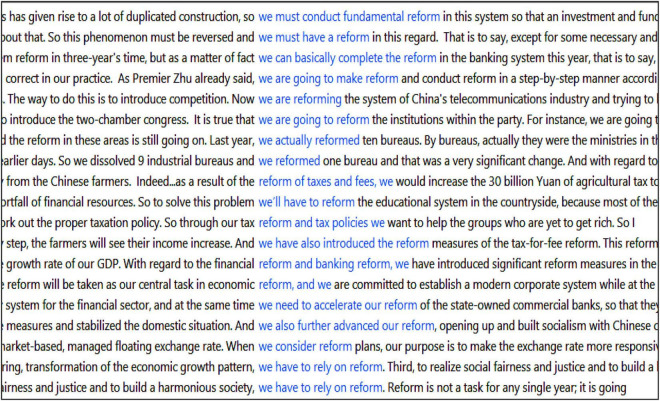
Screenshot of concordance lines featuring reform*, “we” and modality (by collocation).

Comparative analysis shows that self-referential items (“we” and its related forms, “government” and the toponym “China”) are amongst the top collocates in both subcorpora. Interestingly, these self-referential items are rendered considerably more prominent in the TT (234 instances) than the ST (136 instances), constituting a 72% increase. This increased prominence in the interpreted English discourse further highlights the Chinese government’s crucial role as the predominant actor behind China’s various reforms. This is illustrated in Examples 2 and 3. Also, reform*/restructur* in the TT are more closely associated with modality (will, need, must, can, should, have to) than in the ST (104 instances *versus* 78 instances). The more **modalised TT** conveys a stronger level of willingness and commitment in the government’s reform efforts in English (Figure 2 illustrates the close associations of reform* with “we” and/or modality in English).

Regarding actions (verbs), the information has been more or less accurately rendered, including, for instance, what, how and why to pursue, advance, deepen and push forward various reforms. Finally, in terms of propositional content and major concepts, the collocate list suggests that China has been focussing on various reforms (e.g., market-oriented reform, banking reform, SOE reform, rural reform, exchange rate reform and housing reform). Comparisons between the items on both lists illustrate that these miscellaneous reforms are the result of the Chinese STs. This points to a general maintenance of the Chinese discourse.

**FIGURE 3:**
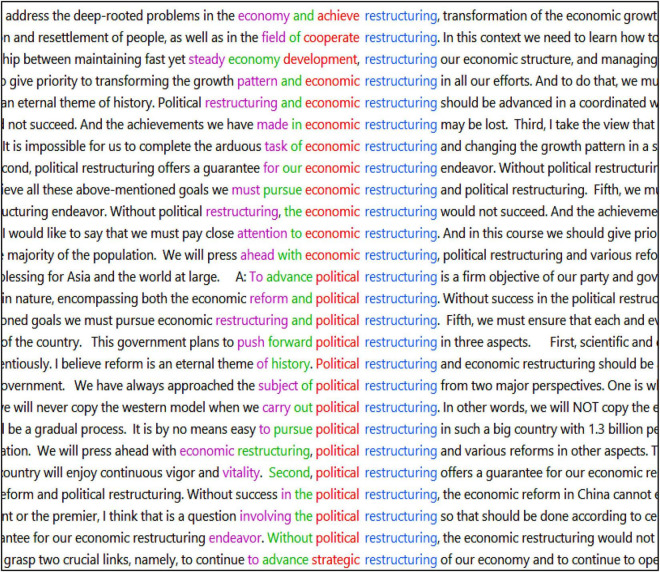
Screenshot of concordance lines featuring economic/political restructuring (sorted to the left).

Although the propositional content has been largely maintained by the interpreters, items relating specifically to China’s system and institutional structure seem to be significantly under-represented in the TT. There are 154 instances of system and institution-related items in the ST (体制86; 制度44; 机构12; 结构7; and 结构性5). However, there are interestingly only 92 instances in the interpreted TT (system/systems 64; structure/structural 19; and institutional 9). This deficit is evidenced visibly in the interpretation of 政治体制改革 and 经济体制改革. While the two constructions are successfully conveyed between the two subcorpora statistically in number, clear shifts in wording were identified. Out of the 21 instances of 政治体制改革 (literally: political system reform) and 13 instances of 经济体制改革 (literally: economic system reform) in the ST, 13 instances (62%) and 9 instances (69.2%) are rendered into English, respectively, as “political restructuring” and “economic restructuring,” without explicitly mentioning “system” (see Figure 3). Examples of this are “reform is an eternal theme of history. Political restructuring and economic restructuring should be advanced in a coordinated way” and “political restructuring offers a guarantee for our economic restructuring endeavour. Without political restructuring, the economic restructuring would not succeed. And the achievements we have made in economic restructuring may be lost” (2011 press conference).

Rather than adopting the obvious strategy of literal translation, which is consistently seen in the treatment of other specialised reforms, the pronounced tendency to translate *political/economic system reform* in Chinese as *political/economic restructuring* is salient ideologically. In the Chinese context, political and economic system reforms appear to be gradual and ameliorative processes underpinned by the upholding of the socialist system. Political reform, as Premier Wen explicates, revolves around “what is socialism, how to build and improve socialism” and “what kind of party to build and how to build it” (2003 press conference). Therefore, fundamental systematic change is “never on the agenda” and the reform process, if anything, is “aimed to re-strengthen the existing political system” (Ploberger, 2016, 72). Similarly, economic system reform in the Chinese context is not a switch toward capitalism but reform predicated on the espousal of “socialist market economy” (*cf*. “Collocational Patterns Relating to the Economy” for more details). Mentions of *political/economic system reform* in Chinese are of relevance and might serve to score domestically in showing the government’s resolve to effect positive changes (e.g., tackle corruption, streamline administration). However, *system reform* in English seems to indicate more drastic changes to mend the dysfunctional or faulty system. The word *restructuring* by comparison places more emphasis on the government’s self-improvement institutionally as if it were a corporation. Arguably, opting for a literal rendition in English potentially risks the incremental economic and political reforms being misconstrued by the outside world as any fundamental changes in China’s (presumably faulty) political and economic systems. As such, the repeated articulations of *political/economic restructuring* can be seen as the interpreters’ conscious decision to accommodate the TT audience and avoid causing misinterpretation and speculation internationally. This level of exceptional and nuanced treatment and (re)contextualisation suggests that the interpreters have aligned with the government and taken into account the international reception and potential global consequences of the ST message (see Example 3). This, as such, points toward the interpreters’ active mediation and (re)construction of Beijing’s version of truth and knowledge.

Moreover, China’s discursive articulation on reform is often closely associated with other important abstract concepts. For example, there are 28 instances of the overarching construction 改革开放 (literally: reform and opening-up) in Chinese and the same number of its rough equivalents in English: *reform and opening-up* (21 instances); *reform, opening-up and* (*socialist*) *modernisation* (*drive*) (4 instances); reform, opening-up and buil* socialism with Chinese characteristics (2 instances); *reform and opening wider* (1 instance). “Reform” is also closely associated with “development” and, to a lesser extent, “stability” in English: *reform and develop* (1 instance), *reform and development* (11 instances) and *reform, development and stability* (2 instances). The juxtaposition of these concepts shows that cognitively reform is inextricably linked with development and stability in China. That is, there cannot be real development without reform. Similarly, without stability as the precondition, reform and development are impossible in China’s reform and opening-up^2^ efforts. These patterned constructions are accurate reflections of the ST.

The examples below illustrate some of the points discussed above (the underlined sections indicate major shifts). Example 2 concerns the interpreter’s additions of self-referential items and the word “reform” as well as the use of the high-modality “must.” Example 3 concerns the interpreter’s additions of self-referential items and the nuanced treatment of “economic/political system reform” in the TT.

**Example 2 (2016)**

**ST:** 所以简政放权必须一以贯之。 哪里遇到问题碰到阻力就要设法去解决。

**Gloss:** So the streamlining of administration and delegation of power must be consistent. Where problems are met and resistance is encountered, efforts need to be made to solve them.

**TT:** We must make persistent efforts to forge ahead with this government reform and wherever there is an obstacle to this reform, the government must get right on it.

**Example 3 (2011)**

**ST:** 而做到所有这一些, 都必须推进经济体制改革和政治体制改革。

**Gloss:** To achieve all these, economic system reform and political system reform must be pushed forward.

**TT:** If we are to achieve all these above-mentioned goals, we must pursue economic restructuring and political restructuring.

Notably, the interpreter-mediated discourse often gains further international currency as it appears, fully or partially, in other texts. For instance, the interpreter’s nuanced wording “political restructuring” was, *inter alia*, used intertextually in the headline of an English-language news report^3^. Similarly, the interpreter-mediated discourse featuring the additions of self-referential items and stronger modality use even appeared on the website of the Chinese embassy in the United States^4^ as an official record. This highlights the significance of the interpreters as vital agents and (re)constructors of Beijing’s sociopolitical “truth” and knowledge beyond China’s national borders and the role of interpreter-mediated discourse as a crucial source of meaning potential (Gu and Wang, 2021) in interlingual and intercultural communication.

## Conclusion and Discussion

To conclude, the meta-narrative of reform and opening-up (ROU) has been an overarching story justifying and legitimating China’s sociopolitical systems and various policies and decisions in post-1978 China as a developing country. This article examined the key concepts constitutive of China’s reform and opening-up metadiscourse (e.g., DEVELOPMENT, ECONOMY, REFORM, MARKET, MODERNISATION, STABILITY, OPENNESS, HARMONY, and SOCIALISM). Comparative analyses overall suggest the interpreters’ general alignment and often increased alignment through their increased (re)production of certain items in English *vis-a-vis* the Chinese ST (a 17.75% increase overall and noticeable increases in most categories). The interpreters’ proliferated mentions of such lexical items constitute a case of repetition (Fairclough, 2000; Beaton, 2007; Lahlali, 2012). This, no doubt, leads to the interpreters’ maintenance and often further reinforcement of China’s reform and opening-up metadiscourse (cf. Example 2 for the repeated additions of “reform”), thus strengthening the government’s institutional hegemony overall. Such (increased) interpreter alignment and (re)construction of Beijing’s discourse can be viewed as cumulative and imperceptible in nature to the general global audiences. This is in line with Fairclough’s observation that “ideology is most effective when its workings are least visible” (1989, 85). Admittedly, the interpreters’ repeated production and use of certain lexical items might be explained by various factors (e.g., the linguistic differences^5^ between Chinese and English). Nevertheless, from a product-oriented perspective and given the crucial far-reaching impact of interpreting into English, this is still highly salient and potentially consequential discursively.

In addition to the overall mediation, attention was focussed on two major constitutive concepts ECONOMY and REFORM at different levels. It is found that China and the government are often given more prominence as the chief social actor and agent in English (cf. Examples 2 and 3). This helps render more explicit and emphatic the indispensable role of the government as a vital actor for China’s reform, development and modernisation and important gatekeeper in safeguarding China’s stability and harmony in its socialist advancement as a developing country. Also, the TT is often rendered more modalised (cf. Example 2), exhibiting a stronger level of commitment, willingness and certitude. Discursively and rhetorically, this strengthens the Chinese original and (re)constructs a more favourable image of the government being present, active, committed and determined in various aspects of China’s reform and opening-up.

Interestingly, China’s ROU discourse is often successfully (re)presented in an intertwined web, appearing in the form of seemingly axiomatic dyads, triads and longer patterns (“reform, opening-up and modernisation,” “socialist market economy,” “economic restructuring,” “reform and development,” “reform, development and stability” *etc.*). These collocations are indicative of the government’s discursive and thinking patterns that reform, economic development, opening-up, modernisation, stability and socialism are inextricably connected and that China’s reform and opening-up efforts are market-oriented yet essentially socialist. That is, despite the increasing openness, development and market-oriented reforms, China’s ROU is not a wholesale embracing of capitalism but a pragmatic approach underscoring Socialism with Chinese characteristics. Cognitively and discursively, the juxtaposition and entanglement of these core concepts lead to the internalising and mutual enhancing of the various elements central to China’s ROU discourse. This, facilitated by the interpreters, renders China’s transition toward the pragmatist market-oriented reform and opening-up more justified, naturalised and convincing in English. In addition, the various condensed overarching discursive formulations (e.g., “socialist market economy”) are well (re)produced, maintained and sometimes added in the interpreted discourse (cf. Example 1). The premiers’ repeated articulations of the ritualised guiding formulations, facilitated by the interpreters, work to further consolidate and seemingly render unchallenged China’s hegemonic discourse.

If the aim of the televised press conferences is to project Beijing’s version of fact and reality and have China’s voice heard as the world’s largest developing country, then the more emphatic, forceful and convincing rendering of the ROU meta-narrative in English leads to a stronger voice for Beijing internationally with far-reaching global ramifications beyond the confines of the press conference room. This is particularly true given the outward-facing and high-profile nature of the discursive event and the fact that the interpreted product is often quoted *verbatim*, further (re)mediated and taken for granted by default as a reliable source of official information. As such, beyond a merely mechanical transference of content between languages, the press conference interpreters’ strengthening of Beijing’s ROU meta-narrative points to their key role in (re)telling China’s story and (re)disseminating sociopolitical truth and knowledge globally. This, in turn, also possesses the potentiality of further contributing to the delicate and constantly shifting power differentials between China and the more dominant and vociferous West, at least discursively. Such interpreter mediation seems particularly interesting, considering that those interpreters tend to further progress in their careers later on as government officials, ambassadors and even China’s foreign ministers. While outwith the scope of the current study, it would certainly be interesting to explore the reasons behind the interpreters’ mediation and amplification of the official message possibly from a sociological perspective and/or drawing on other methodological approaches (e.g., interview). This might help shed light on how (re)creating a better image for China and acting in China’s interests might be related to those interpreters’ career development and progression within the institution as junior political cadres. At the crossroads of interpreting studies, CDA, corpus linguistics, image studies, media and communication studies, this interdisciplinary study contributes to scholarship through illustrating the crucial role of interpreters and interpreting in (re)articulating a developing country’s overarching justificatory discourse and in (re)presenting a viable version of sociopolitical system and developmental path in front of the international community.

## Data Availability Statement

The original contributions presented in this study are included in the article/supplementary material, further inquiries can be directed to the corresponding author.

## Author Contributions

The author confirms being the sole contributor of this work and has approved it for publication.

## Conflict of Interest

The author declares that the research was conducted in the absence of any commercial or financial relationships that could be construed as a potential conflict of interest.

## Publisher’s Note

All claims expressed in this article are solely those of the authors and do not necessarily represent those of their affiliated organizations, or those of the publisher, the editors and the reviewers. Any product that may be evaluated in this article, or claim that may be made by its manufacturer, is not guaranteed or endorsed by the publisher.

## Footnotes

1 An examination of the related notions 市场** and market* in both subcopora shows that great emphasis is placed on market in China’s discursive articulations. Also, “market” is rendered significantly more prominent in English both overall (172 mentions *versus* 227 mentions and hence a 32% increase) and diachronically (51.5, 34.4, and 21.3% increase in each administration). This shows increased interpreter alignment and further strengthening of Beijing’s discourse. Discursively, this conveys an image that Beijing is strongly committed to market-oriented reforms in English. This is a trend consistently found in most of the major themes and concepts identified in Table 2.

2 Given the limited space, the related notion “openness” is briefly explored. While there are 121 instances of 开放/放开/敞开/公开 in Chinese, there are 136 instances of open* (open, opened, opening, opening-up, openness) in English (a 12.4% increase), thus making the government appear more committed to further opening-up in English. Diachronically, this trend is reflected in each administration. There are increasing mentions of “openness” across the three administrations in both languages (2.6, 5.9, and 9.8 times per year in Chinese *versus* 4.2, 5.9, and 11.2 in English). This points to an increasing level of openness as China’s reform and opening-up progresses (where China has transformed from a closed inward-looking country to an increasingly open nation keen to embrace the world). Some of the concordance lines featuring openness include “only a nation that is open and inclusive can prosper,” “China will unswervingly pursue the policy of opening-up to the outside world,” “we will further open up China to the rest of the world,” “we should open the Chinese telecommunications market to foreign investors”.

3
http://www.china.org.cn/china/NPC_CPPCC_2011/2011-03/14/content_22134394.htm

4
http://www.china-embassy.org/eng/zt/000/t1349082.htm

5 The interpreters’ tendency to add “we” might have been partially explained by such linguistic differences. In Chinese, for instance, some sentences may not necessarily have explicitly articulated subjects. As such, interpreters might add subjects in the process.
